# A preliminary investigation of amino acid and acylcarnitine levels in neonates from the Tibet autonomous

**DOI:** 10.3389/fgene.2022.941938

**Published:** 2022-09-26

**Authors:** Chunyan Zhang, Drun Dha, Yuxuan Cheng, Ya Ma, Yan Meng, Drun Tse, Dolma Ngawang, Pedrun Dekyi, Tao Jiang, Yang Shu, Jiayi Cui, Jing Li, Yaping Tian

**Affiliations:** ^1^Birth defect prevention and Control Technology Research Center, Medical Innovation Research Division, Chinese PLA General Hospital, Beijing, China; ^2^Department of Pediatrics, Maternity and children’s Hospital of Tibet Autonomous Region, Tibet, China; ^3^Department of pediatrics, The second people’s hospital of Tibet Autonomous Region, Tibet, China; ^4^Department of Pediatrics, Chinese PLA General Hospital, Beijing, China; ^5^Department of Women and children, Health commission of Tibet autonomous region, Tibet, China; ^6^Department of cardiology, Beijing hospital, Beijing, China

**Keywords:** amino acid, acyl carnitine, newborn screening, Tibet region, reference interval, tandem mass spectrometry

## Abstract

**Background:** The purpose of the study was to investigate the levels of amino acids and acylcarnitines in newborns of the Tibet Autonomous Region for the first time and to provide an experimental basis for the diagnosis of genetic metabolic diseases.

**Methods:** We detected concentrations of 43 kinds of amino acids, acylcarnitines and succinylacetone in the dried blood spots of 18482 newborns using liquid chromatography tandem mass spectrometry and diagnose the case by gene sequencing. We compared the indexes between Tibet and our lab, where most data come from an inland area and Han Chinese people. Then we compared amino acid and acylcarnitine levels of seven regions in Tibet and explored their impact factors.

**Results:** We described the levels of amino acids and acylcarnitines in Tibet newborns using 95% confidence intervals. The distribution of amino acid and acylcarnitines were different in Tibet.

**Conclusion:** This study has contributed to filling in the blanks of Tibet newborn screening, which should be considered in the newborn metabolic disease screening in this area.

## Introduction

Inherited metabolic diseases (IMD) are caused by genetic mutations that interfere with typical metabolism. This genetic mutation tends to result in a deficiency of an enzyme defect, leading to a lack of the enzyme’s products as well as an accumulation of the enzyme’s substrates, which then causes a series of clinical symptoms (Sahai and Marsden, 2009). At present, more than 1,000 types of IMD have been diagnosed (Ferreira and van Karnebeek, 2019). While individual metabolic disorders are rare, collectively, their incidence is approximately 1 in 1,000 (Hendriksz, 2009).

Owing to the high morbidity, mortality and strong risk of recurrence in affected families, neonatal IMD screening is an important element of modern preventive medicine as it allows the identification of both potential and asymptomatic infants as early as possible. Meanwhile, neonatal screening programs are an effective measure to reduce birth defects and improve the health of a population.

At present, the technique liquid chromatography-tandem mass chromatography (LC-MS/MS) has become the ideal analysis technique for IMD screening, due to its high sensitivity, signal-to-noise ratio, high specificity and high selectivity (Rashed et al., 1997) (Tu et al., 2010). In China, Shanghai and Zhejiang were the first to apply LC-MS/MS technology to newborn screening, and it is now being gradually introduced into the public health care network system in China (Gu et al., 2002) (Maitusong et al., 2012).

In 2020, the Chinese National Center for Clinical Laboratories (NCCL) launched a nationwide study to investigate the dynamic pattern of 35 MS/MS NBS biomarkers and establish accurate and robust reference intervals, in which 4,714,089 Chinese neonates were tested in participating centers/laboratories (He et al., 2021). However, there is little data on neonatal IMD screening in the Tibet Autonomous Region. This region is located in the southwest of the Qinghai-Tibet Plateau. With a mean elevation of more than 4,000 m, it is known as the ‘roof of the world’. The region covers an area of 122.84 million square kilometers, which accounts for about one eighth of the total area of China. The Tibet Autonomous Region has seven prefecture-level cities and had a population of approximately 3,656,000, a birth rate of 14.6‰ and around 53,400 newborns in 2020. Statistics from the population and family planning commission of the region in 2018 showed that the incidence of birth defects in Tibet was 10.2%, which was significantly higher than the national average 5.6% (Cui et al., 2015) (Zhuoma Solang, 2018). Among the permanent residents in Tibet Autonomous Region, 90.48% are Tibetan, 1.35% are other ethnic minorities, and 8.17% are Han nationality. Due to the special geographical environment and nomadic life style in Tibet, a special diet culture of Tibetan people has been formed, mainly highland barley and beef and mutton, with a single structure and few vegetables. At the same time, the newborn diet has special customs, including eating highland barley wine and yak milk after birth, and the Han people mainly use breast milk or milk powder. Therefore, the metabonomics of newborns in this area is relatively special. The ‘birth defect intervention project’ was started in this region in November 2015, meaning that neonatal IMD screening was carried out there for the first time.

We performed the detection and statistics of amino acid and acylcarnitine levels in neonates from the Tibet Autonomous, with the aim of providing a preliminary reference data in this region.

## Materials and methods

### Patients and samples collection

Samples were taken from 18482 live born infants from seven provinces of Tibet Autonomous Region between October 2015 and February 2021. Heel blood was collected after 72 h after the infants were born and fully breastfed, and then dropped onto filter paper (Whatman 903). Blood spot samples were placed in the shade to dry and were sealed and stored at -4°C prior to detection.

### LC-MS/MS detection

Small molecule metabolites of dry blood spots were extracted using underivatized amino acids, carnitine and succinylacetone assay kits (PerkinElmer, Finland) according to the manual. Concentrations of 43 types of amino acid, carnitine and succinylacetone were detected by liquid chromatography and tandem mass spectrometry (Xevo TQ Detector, Waters, Milford, United States) and then analyzed by the software Masslynx (Waters Corporation, Milford, United States) and Neolynx. (Waters Corporation, Milford, United States).

### Disease diagnose

The diseases were diagnosed by combining with clinical manifestation, laboratory examination and gene sequencing. Genomic DNA of peripheral blood leukocytes from patients and their parents was extracted using a QIAamp DNA Blood Mini Kit (Qiagen, Hilden, Germany). The genes of patients were sequencing by next-generation sequencing technology (Illumina Exome Panel, Illumina, United States), then the mutations of the parents were confirmed by Sanger sequencing. The data was analyzed and reported by software TGex (tgex.genecards.org).

### Statistical analysis

Between-group comparisons were performed by independent-samples t-tests and ANOVA with Bonferroni posthoc multiple comparisons test for normally distributed data or Mann-Whitney U test for non-parametric sample distribution. All statistical analysis was performed using SPSS (version 21.0), and two-tail *p* < 0.05 was considered statistically significant. Graphs were generated using GraphPad Prism software (version 6.0).

### Ethics

This study was approved by the Ethics Committee of the Chinese PLA General Hospital (No. S2018-025-01) and this trial has been verified by Chinese Clinical Trial Registry (No. ChiCTR1800016903). All participants provided informed written consent for sample collection, as well as permission for the samples’ use in research.

## Results

### The distribution of neonates from the tibet autonomous region

A total of 18482 neonatal screenings were conducted between 2015 and 2021, including 10719 males and 7,763 females. Some 99.67% of these babies (18421/18482) were Tibetan. The cases were distributed in seven prefecture-level cities in the Tibet Autonomous Region, including 6,586 in Lhasa, 2,850 in Naqu, 1,155 in Changdu, 1,079 in Shannan, 5,629 in Xigaze, 593 in Ngari and 590 in Nyingchi. The distribution of neonates from the Tibet Autonomous Region is depicted in Table 1.

**TABLE 1:** Distribution of neonates from the Tibet Autonomous Region.

District	Total	Gender		Gestational age		Birth weight	
		Male, *n*/%	Female, *n*/%	Average	95%CI	Average	95%CI
Lhasa	6,586	3,859/59	2,727/41	39.58	39.53–39.63	3,202.32	3,174.96–3,229.67
Ngari	593	356/60	237/60	39.76	39.66–39.87	3,231.81	3,070.22–3,393.40
Xigaze	5,629	3,329/59	2,300/41	39.23	39.18–39.27	3,291.78	3,257.51–3,326.05
Nyingchi	590	366/62	224/38	39.05	38.74–39.36	3,224.5	3,127.11–3,321.90
Changdu	1,155	650/56	505/44	39.45	39.38–39.52	3,408.72	3,345.79–3,471.66
Shannan	1,079	589/54	490/46	39.83	39.78–39.89	3,178.86	3,100.46–3,257.26
Naqu	2,850	1,570/55	1,280/45	39.28	39.19–39.36	3,468.35	3,406.55–3,530.14
Total	18482	10719/58	7,763/42	39.39	39.36–39.41	3,322.69	3,301.75–3,343.63

### Comparison to the accumulated laboratory results

**FIGURE 1:**
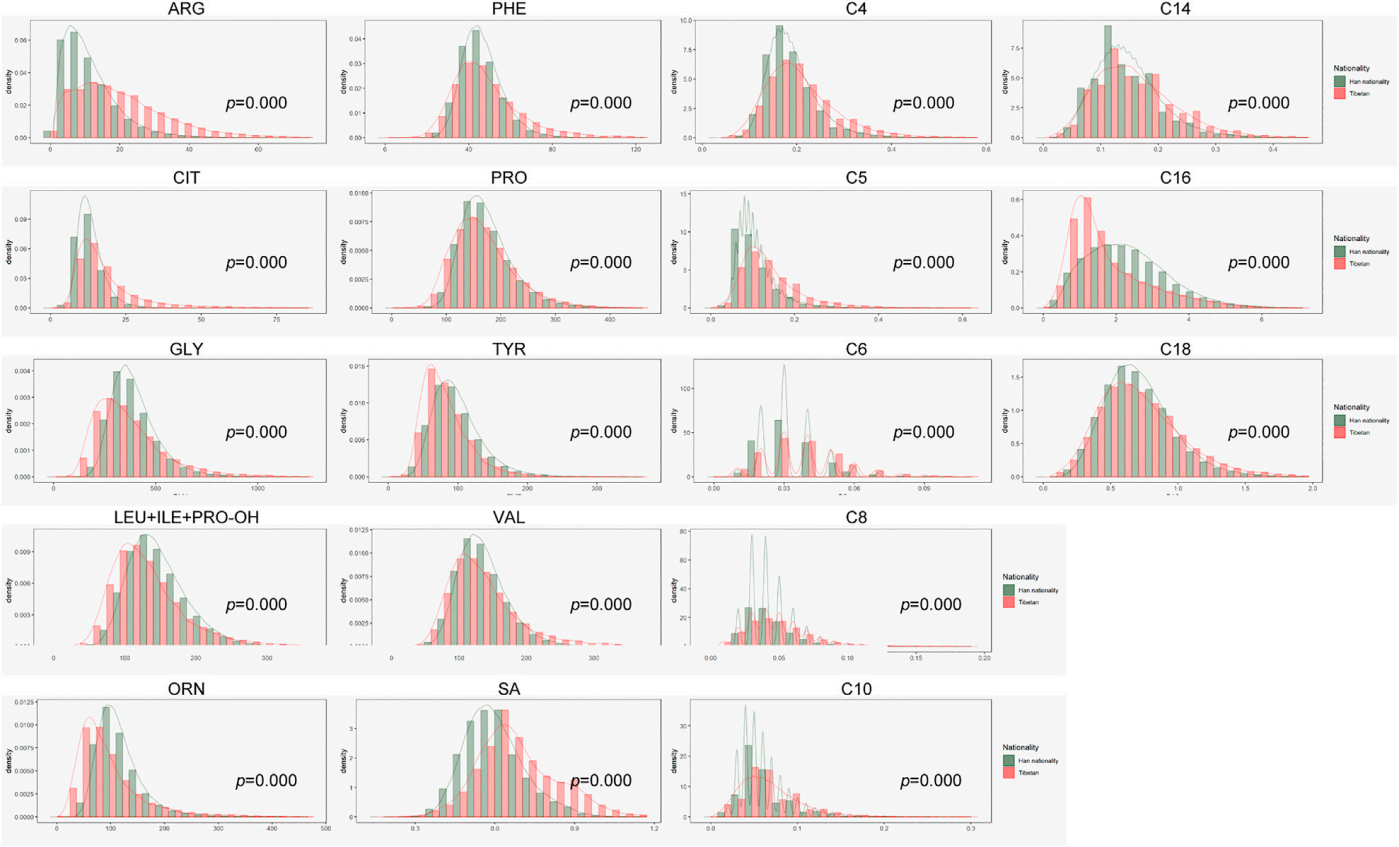
Comparison of data between samples from Tibet and our laboratory.

Before this study, there was little data on neonatal screening from the Tibet region and Tibetan people. As shown in Figure 1, concentration of many indexes in the Tibet region were different from our lab, where most data come from inland areas and Han Chinese people. Amino acids, ketones indexes, Arg, Phe, Cit and SA were higher, while Orn, Pro, Tyr, Gly, Leu + Ile + Pro-OH and Val were lower in Tibet than in inland areas. The concentration of medium and long chain acylcarnitine C6-C18 were higher than in inland areas. At the initial stage, we used the original cut-off value for screening, and the recall rate in Tibet was close to 6%. Obviously, the inland reference range was not very suitable for screening in Tibet, which would cause a higher false positive rate.

### Description of levels of amino acids and acylcarnitines in tibet neonates

The levels of amino acids and acylcarnitines in neonates from the Tibet Autonomous Region were described based on the P0.5%∼P99.5% of the values (Table 2 and Supplemental Table S1). If we recall refer to these values, the recall rate of statistical screening was significantly reduced to 2.35%. These results, which can be seen in Table 3, are approximate to the recall rates of other regions. Based on the results of metabolite and gene detection, we diagnosed four cases with phenylketonuria (PKU) (Table 4). Among of the four PKU cases, case 1 and case 2 belong to the same family.

**TABLE 2:** Statistical results of amino acids and acylcarnitines of neonates from the Tibet Autonomous Region.

Index	Average (μmol/L)	Percentage (*p*)							
		*p*,0.001	*p*,0.005	*p*,0.01	*p*,0.5	*p*,0.95	*p*,0.99	*p*,0.995	*p*,0.999
ALA	305.596	82.535	119.670	133.826	286.270	501.280	680.896	819.484	1,006.552
ARG	19.478	1.275	1.530	1.768	16.620	44.100	63.992	74.610	110.094
CIT	17.608	3.704	5.314	5.858	14.060	39.610	70.698	91.482	124.694
GLY	368.356	84.140	115.420	129.562	331.280	694.940	1,021.710	1,301.044	1,629.344
LEU + ILE + PRO-OH	131.348	29.892	45.208	52.514	120.910	230.340	314.566	378.943	453.288
MET	13.743	1.650	2.284	2.648	12.690	25.580	34.446	39.995	57.794
ORN	92.749	10.574	17.114	20.398	73.700	216.260	366.072	483.767	635.636
PHE	48.805	11.425	19.214	21.550	45.430	80.530	109.216	135.691	178.446
PRO	170.659	35.407	62.218	71.768	161.510	279.190	358.966	394.431	493.155
SA	0.697	0.290	0.350	0.390	0.680	0.950	1.070	1.130	1.265
TYR	84.976	17.734	28.692	32.286	77.830	151.660	204.806	230.735	308.150
VAL	132.009	32.566	48.710	54.680	119.950	237.330	331.622	395.510	456.814
C0	29.945	4.508	7.404	9.028	28.080	52.470	67.470	76.868	90.719
C2	12.848	0.395	0.770	1.030	11.560	27.890	37.882	38.343	51.997
C3	1.545	0.090	0.190	0.250	1.370	3.210	4.510	4.992	6.590
C3DC + C4OH	0.081	0.010	0.010	0.010	0.070	0.190	0.280	0.310	0.435
C4	0.209	0.060	0.070	0.080	0.190	0.350	0.480	0.550	0.755
C4DC + C5OH	0.206	0.050	0.070	0.080	0.190	0.360	0.480	0.570	0.650
C5	0.135	0.020	0.030	0.040	0.120	0.270	0.400	0.501	0.625
C5:1	0.008	0.000	0.000	0.000	0.010	0.010	0.020	0.030	0.035
C5DC + C6OH	0.122	0.020	0.030	0.040	0.110	0.210	0.290	0.330	0.430
C6	0.040	0.010	0.010	0.010	0.040	0.070	0.090	0.100	0.150
C6DC	0.073	0.020	0.020	0.030	0.070	0.130	0.190	0.221	0.440
C8	0.049	0.010	0.010	0.010	0.050	0.090	0.130	0.140	0.230
C8:1	0.129	0.010	0.020	0.020	0.120	0.270	0.390	0.450	0.550
C10	0.069	0.010	0.010	0.010	0.060	0.140	0.200	0.211	0.320
C10:1	0.053	0.010	0.010	0.010	0.050	0.110	0.150	0.160	0.200
C10:2	0.013	0.000	0.000	0.000	0.010	0.020	0.040	0.050	0.080
C12	0.061	0.010	0.010	0.010	0.050	0.130	0.210	0.230	0.380
C12:1	0.043	0.000	0.010	0.010	0.030	0.110	0.170	0.181	0.300
C14	0.165	0.020	0.030	0.040	0.150	0.300	0.390	0.410	0.535
C14:1	0.056	0.010	0.010	0.010	0.050	0.120	0.200	0.210	0.320
C14:2	0.014	0.000	0.000	0.000	0.010	0.030	0.030	0.040	0.060
C14OH	0.008	0.000	0.000	0.000	0.010	0.020	0.020	0.020	0.030
C16	1.917	0.160	0.290	0.360	1.560	4.260	5.420	5.272	7.026
C16:1	0.111	0.010	0.010	0.020	0.080	0.280	0.370	0.370	0.480
C16:1OH	0.093	0.010	0.020	0.020	0.080	0.210	0.300	0.360	0.450
C16OH	0.015	0.000	0.000	0.010	0.010	0.030	0.040	0.040	0.050
C18	0.729	0.100	0.150	0.188	0.690	1.290	1.670	1.870	2.145
C18:1	1.162	0.145	0.250	0.310	1.110	1.970	2.440	2.571	3.071
C18:1OH	0.022	0.000	0.000	0.010	0.020	0.040	0.050	0.060	0.070
C18:2	0.209	0.020	0.040	0.040	0.190	0.420	0.550	0.601	0.830
C18OH	0.007	0.000	0.000	0.000	0.010	0.010	0.020	0.020	0.020

**TABLE 3:** Statistics of recall rate in Tibet refer to the P5%∼95% values.

District	MS/MS results (μmol/L)			
	Nornal	*n*/%	Abnornal	*n*/%
Lhasa	6,524	99.06	62	0.94
Naqu	2,747	96.39	103	3.61
Changdu	1,102	95.41	53	4.59
Shannan	1,063	98.52	16	1.48
Xigaze	5,502	97.74	127	2.26
Ngari	581	97.98	12	2.02
Nyingchi	581	98.47	9	1.53
Total	18100	97.65	382	2.35

**TABLE 4:** Diagnosed cases in neonatal screening in Tibet.

Case	MS/MS results	Urine organic acid test	Gene detection	Disease
Case 1	C16:1OH = 0.14,ARG = 46.93, (C16 + C18:1)/C2 = 0.09,PHE/TYR = 18.56,CIT/PHE = 0.01,C4 = 0.47,CIT = 36.6,MET = 54.43,PHE = 3,060.9,VAL = 367.61,C3 = 4.6,C14 = 0.42, (LEU + ILE + PRO-OH)/PHE = 0.09, VAL/PHE = 0.12,MET/PHE = 0.02.	The level of phenylacetic acid, phenyllactic acid, Phenylpyruvate, 4-hydroxyphenyllactic acid and 4-hydroxyphenylpyruvate increased	PAH: c.728G>A (*p*.R243Q); PAH: c.1068C>A (*p*.Y356*)	phenylketonurics
Case 2(Case 1 sibling)	C16:1OH = 0.14,ARG/ORN = 0.42,PHE/TYR = 34.18,CIT/PHE = 0.01,PHE = 1867.38,C4DC + C5OH = 0.37, (LEU + ILE + PRO-OH)/PHE = 0.08, VAL/PHE = 0.11,MET/PHE = 0.01.	The level of phenylacetic acid, phenyllactic acid, Phenylpyruvate, 4-hydroxyphenyllactic acid and 4-hydroxyphenylpyruvate increased	PAH: c.728G>A (*p*.R243Q); PAH: c.1068C>A (*p*.Y356*)	phenylketonurics
Case 3	PHE = 585.08,ALA = 762.64,TYR = 276.97,PHE/TYR = 2.11, (LEU + ILE + PRO-OH)/PHE = 0.36, MET/PHE = 0.06,CIT/PHE = 0.03,ARG/PHE = 0.02,ORN/PHE = 0.23.	The level of phenylacetic acid and phenyllactic acid increased; 4-hydroxy-phenyllactic acid index increased; The indexes of uracil and whey acid increased	PAH: c.536C>T (*p*.R176X); PAH: c.1054G>T (*p*.G352C)	phenylketonurics
Case 4	PHE = 1,292.8,ARG/PHE = 0.02,CIT/PHE = 0.03, (LEU + ILE + PRO-OH)/PHE = 0.15, MET/PHE = 0.01, PHE/TYR = 13.93, VAL/PHE = 0.14.	The level of phenylacetic acid, phenyllactic acid and Phenylpyruvate increased	PAH: c.694C>T; PAH: c.694C>T	phenylketonurics

### The effects of diet and altitude on small molecule metabolites

The screening area covers seven prefecture-level cities. A total of 99.67% of the babies (18421/18482) were Tibetan. Due to local limited medical conditions and nomadic customs, the mean blood collection time was 30 days after birth. Most babies had been given supplemental food from the birth, according to the local custom, which caused differences of small metabolites molecules between this area and inland areas. For example, babies in Xigaze region were fed by breast milk and zanba (highland barley) and were also given a small amount of barley wine and buttered tea. Therefore, we initially explored the impact of the special dietary habits in different areas of Tibet on the small molecule metabolites indicators.

**FIGURE 2:**
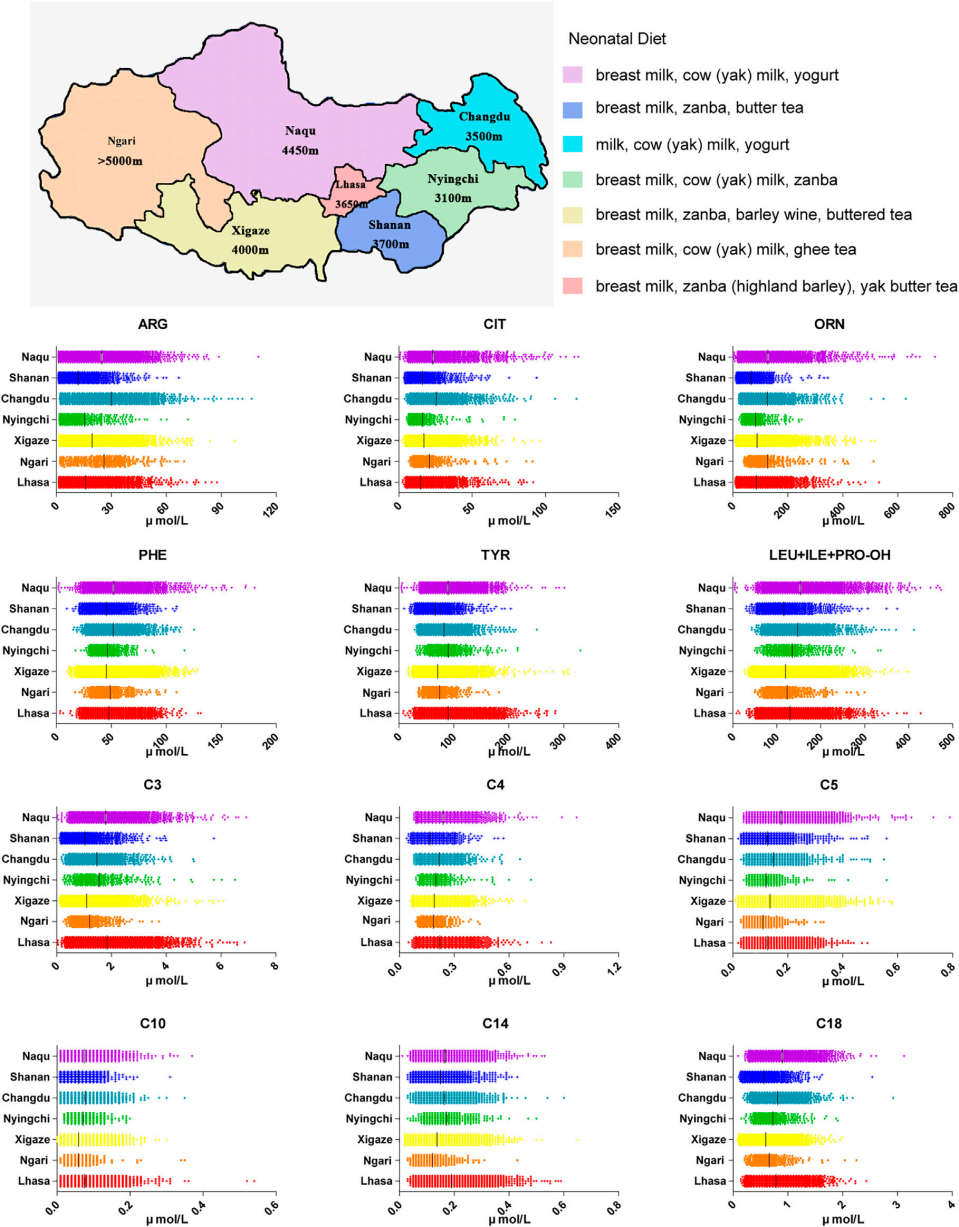
Neonatal diet in Tibet Autonomous Region as well as amino acids and acylcarnitine in different areas of Tibet.

As shown in Figure 2, many amino acids were significantly higher in Changdu and Naqu, where babies were fed with breast milk, cow (yak) milk and yoghurt, than in Lhasa, Xigaze and Shannan, where babies were fed with breast milk, zanba and yak butter tea. These results were replicated for lipometabolism levels. Although there was no significant difference in free carnitine C0 in the different regions, levels of short-chain acylcarnitine C2-C5 were higher in Changdu and Naqu and were lower in Ngari, Xigaze and Shannan. Levels of medium and long chain acylcarnitine C6-C18 were significantly higher in Lhasa, Changdu, Nyingchi and Naqu than in Ngari, Xigaze and Shannan.

In order to explore effects of climate and altitude on tandem results, we compared the results of Nyingchi and Ngari, which have similar diets. Ngari is located in the southwest border of China and has a mean altitude of more than 5,000 m. Due to its high altitude, the climate is cold and dry, annual rainfall is relatively low, the difference between day and night temperature is large, and winter is long and cold. In contrast, Nyingchi, which is located in the southeast of Tibet, has a mean altitude of 3,100 m, while its lowest point is only 900 m, lower than other areas of Tibet. Due to the Indian ocean current, the climate is warm and comfortable. Annual rainfall is approximately 650 mm, the average annual temperature is 8.7°C, average annual sunshine is 2022.2 h, and the frost-free period is 180 days. Therefore, while the two regions have similar population composition and eating habits, their altitudes, climates and other factors are different.

**FIGURE 3:**
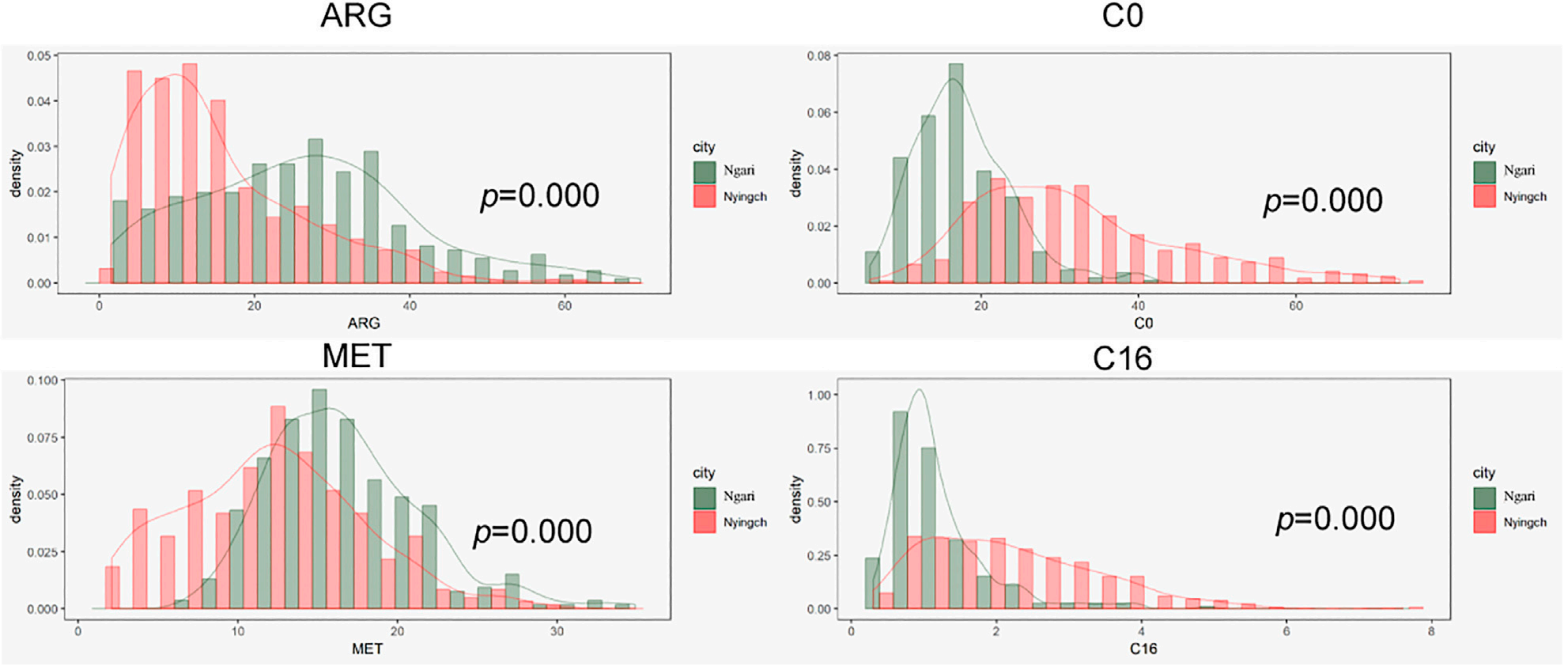
Comparison of metabolite index between Ngari and Nyingchi.

As shown in Figure 3, amino acids that easily degraded were lower in Nyingchi and higher in Ngari region, such as Arg and Met. These results were consistent with previous reports that Arg and Met would fluctuate regularly, with low concentration in summer and high concentration in winter. Most acylcarnitine levels in the Ngari region were lower in Ngari, which may be related to the low basal metabolic rate in Ngari region.

## Discussion

In the past 10 years, MS/MS was widely used for neonatal hereditary metabolic disease screening in China, improving the ability and efficiency of newborn screening (Huang et al., 2017). The NCCL initiated a large-scale NBS survey where 114 newborn screening laboratories/centers distributed in 29 provinces or municipalities participated in 2020, and 59 of the 114 (50.9%) MS/MS NBS facilities developed their own reference intervals based on their local samples (He et al., 2021). Until now, there was little data relating to the screening, diagnosis and monitoring of neonatal diseases in the Tibet Autonomous Region. This was for many reasons, such as nomadic and technological factors. Statistics from the population and family planning commission of the region in 2013 showed that incidences of birth defects in Tibet were significantly higher than the national mean. Therefore, early screening as well as timely and effective treatment are crucial for protecting the healthy growth of newborns from this area. Small molecular metabolites reflect the conditions of newborns and mothers, while regional differences in dietary habits, ethnic groups, climate and incidence rates lead to regional differences in indicators. Therefore, it is necessary to establish a reference range of the local population, as this can reduce the false positive rate and improve accuracy.

Here we investigated the amino acid and acylcarnitine levels in 18482 neonates from the Tibet Autonomous Region, providing preliminary data in this region for the first. There were some unsatisfactory factors, such as the time of blood collection and follow up. According to the technical specifications for neonatal disease screening (2010 version), blood sampling time is 72 h after birth, within 7 days, and full breastfeeding. However, in the actual situation, due to the inconvenient transportation, limited medical conditions, nomadic habits, special eating habits, and weak awareness of medical screening, the blood collection time in Tibet could not meet the requirements of the standard, with an average of 30 days. Therefore, the sampling time was one factor that affect the levels of small molecule metabolites. Considering the particularity of Tibet and the blank of data, we think it is very necessary to set up a new cut-off value according to the actual situation.

Our data were mostly obtained from domestic provinces, including Beijing, Guizhou, Sichuan, Hubei and so on. Compared with inland newborn, the levels of Arg, Cit were higher and the level of Orn was lower in Tibetan newborn. The three amino indicators are related to urea cycle disorder and citrin deficiency. In the urea cycle, Cit reacts with Asp to form arginosuccinate, which is decomposed to Arg; Arg is hydrolyzed to Orn and urea under the action of arginase (Ali et al., 2018) (Kiykim et al., 2018). The upstream and downstream metabolites of Arg were Cit and Orn, respectively. In the analysis of individual data, the three indicators did have similar trends, and the gene detection results for the cases with1.5∼2 times higher levels were negative. Therefore, these results were caused by metabolism *in vivo* and reflected the metabolic level in Tibetan newborns, which may be caused by special diet during newborn, such as high-protein food, like yak milk. Interestingly, in the metabolism pathway of Phe and Tyr, Tibetan healthy newborn showed a higher level of Phe, and lower level of Tyr. Phe, one of special indexes for diagnosing hyperphenylalaninemia, had a maximum value (95%CI) of 130 μmol/L in Tibet, which is higher than our regular cutoff 120 μmol/L (Li et al., 2015) (Liu et al., 2017). The results in Table 4 showed that the level of Phe in patients with PKU were much higher than the normal reference range. The phenomenon maybe related to folate metabolism. It has been reported that a folate-increasing allele of the SNP rs1801133 at the MTHFR locus has an increased frequency in the Tibetan population, more than in Han population, which is possibly a consequence of adaptation to high UV radiation (Yang et al., 2017). To illuminate the phenomenon, further research needed to be done. In terms of lipid metabolism, we observed that most acylcarnitine levels, especially medium and long chain acylcarnitine C6-C18, were higher than in inland area. C0 levels in babies from Tibet babies were lower than others. Newborns from the inland area may have a single diet source (milk or milk powder), but newborns in Tibet, due to local customs, have a varied diet in the first 3 days. The distribution of amino acid and acylcarnitine was different in Tibet and reflect the difference of human metabolism activity in different regions.

We detected accuracy and inaccuracy before we performed the study to ensure that results were reliable. Our laboratory reported results of the neonatal genetic metabolic disease screening (tandem mass spectrometry technology) to the National Center for Examination and took part in interventricular quality assessment twice every year, which ensures that results are valid. The preliminary reference range is only applicable to the current neonatal disease screening in Tibet. After the popularization of the new screening education, the improvement of medical conditions, more standardized blood collection and the expansion of sample size, more suitable cutoff value is required in the future.

## Conclusion

By examining data from more than 18,000 newborns, this study investigated the amino acid and acylcarnitine levels in neonates of the Tibet Autonomous for the first time. Data reflect the metabolic level of newborns in Tibet and fill in gaps in the region. This study suggested an independent cut-off value of neonatal screening should be established in different regions, especially in high altitude areas, which will greatly improve the diagnostic efficiency for neonatal screening.

## Data Availability

The data presented in the study are deposited in the National Population Health Science Data Center repository (https://www.ncmi.cn/), doi:10.12213/11.A005Y.202109.1871.V1.0.

## Glossary

IMD: Inherited metabolic diseases
Ala: Alanine
Arg: Arginine
Cit: Citrulline
Gly: glycine
Leu: Leucine
Ile: isoleucine
Pro-OH: hydroxyprolin
Met: methionine
Orn: ornithine
Phe: phenylalanine
Pro: proline
Asp: aspartate
SA: Succinyl acetone
C0: free carnitine
C2: Acetyl carnitine
C3: Propionyl carnitine
C3-DC: Malonyl carnitine
C4: Butyryl/isobutyryl carnitine
C4-DC: Methylmalonyl/succinyl carnitine
C5:1: Tiglyl carnitine;
C5: Isovaleryl carnitine;
C5-DC: Glutaryl carnitine;
C5-OH: 3-Hydroxy-isovaleryl carnitine
C6: Hexanoyl carnitine
C6-DC: Adipoyl carnitine
C8:1: Octenoyl carnitine
C8: Octanoyl carnitine
C10:1: Decenoyl carnitine
C10: Decanoyl carnitine
C12:1: Dodecenoyl carnitine
C12: Dodecanoyl carnitine
C12-OH: 3-Hydroxydodecanoyl carnitine
C14:2: Tetradecadienoyl carnitine
C14:1: Tetradecenoyl carnitine
C14: Tetradecanoyl carnitine
C14:1-OH: 3-Hydroxy-tetradecenoyl carnitine
C14-OH: 3-Hydroxy-tetradecanoyl carnitine
C16:1: Palmitoleoyl carnitine
C16: Palmitoyl carnitine
C16:1-OH: 3-Hydroxy-palmitoleoyl carnitine
C16-OH: 3-Hydroxy-palmitoyl carnitine
C18:2: Linoleyl carnitine
C18:1: Oleoyl carnitine
C18: Stearoyl carnitine
C18:2-OH: three- Hydroxy-linoleyl carnitine
C18:1-OH: 3-Hydroxy-oleoyl carnitine
