# Supplementary material for: A preliminary investigation of amino acid and acylcarnitine levels in neonates from the Tibet autonomous
Source: Front Genet. 2022 Sep 26;13:941938. doi: 10.3389/fgene.2022.941938 (PMC9589887; doi:10.3389/fgene.2022.941938)
Supplement: Supplementary file 1 [file Table1.docx]

**Supplemental Table. Statistical results of Indicator ratio of neonates from the Tibet Autonomous Region**

| **Index** | **Average (μmol/L)** |  | | | **Percentage(P)** | | | | | |
| --- | --- | --- | --- | --- | --- | --- | --- | --- | --- | --- |
|  |  | P,0.001 | P,0.005 | P,0.01 | | P,0.5 | P,0.95 | P,0.99 | P,0.995 | P,0.999 |
| C0/(C16+C18) | 13.996 | 2.406 | 3.095 | 3.445 | | 12.306 | 28.689 | 38.428 | 43.619 | 53.425 |
| C0/C16 | 21.056 | 3.014 | 3.874 | 4.334 | | 18.658 | 44.625 | 58.711 | 66.860 | 85.215 |
| C0/C18 | 46.202 | 10.570 | 13.554 | 15.065 | | 41.135 | 89.350 | 125.535 | 147.191 | 178.528 |
| C3/C0 | 0.056 | 0.006 | 0.010 | 0.011 | | 0.049 | 0.122 | 0.166 | 0.181 | 0.233 |
| C3/C2 | 0.150 | 0.031 | 0.041 | 0.046 | | 0.123 | 0.316 | 0.565 | 0.801 | 1.169 |
| C3/C16 | 0.947 | 0.117 | 0.196 | 0.237 | | 0.811 | 1.982 | 2.928 | 3.453 | 4.746 |
| (C3DC+C4OH)/C4 | 0.395 | 0.053 | 0.075 | 0.091 | | 0.344 | 0.826 | 1.211 | 1.375 | 1.838 |
| (C3DC+C4OH)/C8 | 1.719 | 0.216 | 0.333 | 0.400 | | 1.500 | 3.333 | 4.884 | 6.000 | 7.883 |
| (C3DC+C4OH)/C10 | 1.273 | 0.167 | 0.250 | 0.294 | | 1.125 | 2.500 | 3.667 | 4.500 | 6.500 |
| (C3DC+C4OH)/C16 | 0.050 | 0.007 | 0.010 | 0.011 | | 0.040 | 0.121 | 0.190 | 0.222 | 0.280 |
| C4/C2 | 0.025 | 0.005 | 0.006 | 0.006 | | 0.017 | 0.066 | 0.128 | 0.201 | 0.326 |
| C4/C3 | 0.168 | 0.042 | 0.050 | 0.056 | | 0.146 | 0.323 | 0.500 | 0.708 | 1.238 |
| C4/C8 | 4.975 | 1.020 | 1.400 | 1.566 | | 4.333 | 10.000 | 15.000 | 19.000 | 24.534 |
| (C4DC+C5OH)/C0 | 0.008 | 0.002 | 0.002 | 0.003 | | 0.007 | 0.014 | 0.019 | 0.022 | 0.028 |
| (C4DC+C5OH)/C3 | 0.176 | 0.031 | 0.038 | 0.042 | | 0.142 | 0.398 | 0.591 | 0.755 | 0.976 |
| (C4DC+C5OH)/(C5DC+C6OH) | 1.924 | 0.426 | 0.583 | 0.643 | | 1.636 | 4.000 | 5.750 | 7.500 | 9.104 |
| (C4DC+C5OH)/C8 | 5.067 | 0.732 | 1.091 | 1.273 | | 4.273 | 11.000 | 17.000 | 22.092 | 30.000 |
| C5/C0 | 0.005 | 0.001 | 0.001 | 0.002 | | 0.004 | 0.010 | 0.015 | 0.020 | 0.026 |
| C5/C2 | 0.017 | 0.002 | 0.003 | 0.003 | | 0.011 | 0.051 | 0.101 | 0.155 | 0.241 |
| C5/C3 | 0.108 | 0.021 | 0.027 | 0.029 | | 0.090 | 0.232 | 0.359 | 0.474 | 0.753 |
| C5/C8 | 3.294 | 0.500 | 0.667 | 0.778 | | 2.667 | 7.550 | 12.390 | 16.364 | 20.767 |
| (C5DC+C6OH)/C0 | 0.005 | 0.001 | 0.001 | 0.001 | | 0.004 | 0.009 | 0.012 | 0.013 | 0.018 |
| (C5DC+C6OH)/(C3DC+C4OH) | 2.033 | 0.320 | 0.421 | 0.478 | | 1.625 | 4.750 | 7.500 | 10.000 | 15.000 |
| (C5DC+C6OH)/(C4DC+C5OH) | 0.654 | 0.110 | 0.148 | 0.174 | | 0.611 | 1.200 | 1.556 | 1.709 | 2.349 |
| (C5DC+C6OH)/C8 | 2.916 | 0.545 | 0.727 | 0.833 | | 2.500 | 6.000 | 9.500 | 12.000 | 17.000 |
| (C5DC+C6OH)/C16 | 0.086 | 0.016 | 0.020 | 0.022 | | 0.069 | 0.200 | 0.328 | 0.422 | 0.586 |
| C6/C3 | 0.004 | 0.001 | 0.001 | 0.001 | | 0.003 | 0.011 | 0.019 | 0.024 | 0.043 |
| C8/C2 | 0.006 | 0.001 | 0.001 | 0.001 | | 0.004 | 0.016 | 0.033 | 0.055 | 0.097 |
| C8/C3 | 0.041 | 0.004 | 0.007 | 0.008 | | 0.033 | 0.094 | 0.156 | 0.200 | 0.305 |
| C8/C10 | 0.754 | 0.200 | 0.400 | 0.444 | | 0.727 | 1.000 | 1.333 | 2.000 | 2.000 |
| C10/C3 | 0.057 | 0.006 | 0.008 | 0.010 | | 0.045 | 0.136 | 0.220 | 0.273 | 0.430 |
| C10:2/C10 | 0.234 | 0.000 | 0.000 | 0.000 | | 0.200 | 0.500 | 1.000 | 1.000 | 2.000 |
| C12/C8 | 1.259 | 0.270 | 0.400 | 0.500 | | 1.200 | 2.167 | 2.875 | 3.000 | 4.000 |
| C12/C10 | 0.912 | 0.255 | 0.333 | 0.357 | | 0.857 | 1.500 | 2.000 | 2.000 | 2.349 |
| C14/C3 | 0.129 | 0.027 | 0.034 | 0.039 | | 0.111 | 0.256 | 0.381 | 0.474 | 0.717 |
| C14:1/C2 | 0.006 | 0.001 | 0.001 | 0.001 | | 0.004 | 0.016 | 0.033 | 0.057 | 0.113 |
| C14:1/C6 | 1.493 | 0.286 | 0.400 | 0.500 | | 1.333 | 3.000 | 4.333 | 5.333 | 8.000 |
| C14:1/C8 | 1.203 | 0.250 | 0.333 | 0.400 | | 1.000 | 2.250 | 3.000 | 3.250 | 4.147 |
| C14:1/C8:1 | 0.570 | 0.067 | 0.083 | 0.095 | | 0.438 | 1.500 | 2.447 | 3.000 | 4.130 |
| C14:1/C12:1 | 1.445 | 0.429 | 0.540 | 0.625 | | 1.333 | 2.200 | 3.000 | 3.000 | 4.000 |
| C14:1/C14 | 0.361 | 0.069 | 0.086 | 0.098 | | 0.333 | 0.682 | 0.909 | 1.000 | 1.446 |
| C14:1/C16 | 0.033 | 0.008 | 0.009 | 0.011 | | 0.029 | 0.063 | 0.095 | 0.109 | 0.166 |
| C14:1/C18:1 | 0.050 | 0.011 | 0.014 | 0.015 | | 0.043 | 0.104 | 0.159 | 0.190 | 0.254 |
| C16/C2 | 0.208 | 0.029 | 0.038 | 0.043 | | 0.153 | 0.448 | 1.057 | 2.046 | 4.826 |
| C16/C3 | 1.427 | 0.211 | 0.295 | 0.342 | | 1.234 | 2.866 | 4.224 | 5.146 | 8.581 |
| C16OH/C3 | 0.013 | 0.000 | 0.000 | 0.002 | | 0.011 | 0.029 | 0.048 | 0.064 | 0.111 |
| C16OH/C14 | 0.104 | 0.000 | 0.000 | 0.031 | | 0.095 | 0.200 | 0.286 | 0.378 | 0.500 |
| C16OH/C16 | 0.010 | 0.000 | 0.000 | 0.003 | | 0.009 | 0.020 | 0.030 | 0.042 | 0.063 |
| C18/C3 | 0.586 | 0.097 | 0.128 | 0.154 | | 0.500 | 1.200 | 1.833 | 2.168 | 3.236 |
| C18OH/C3 | 0.005 | 0.000 | 0.000 | 0.000 | | 0.005 | 0.015 | 0.023 | 0.029 | 0.046 |
| C18OH/C18 | 0.010 | 0.000 | 0.000 | 0.000 | | 0.011 | 0.022 | 0.029 | 0.033 | 0.040 |
| (C16+C18:1)/C2 | 0.342 | 0.066 | 0.082 | 0.089 | | 0.253 | 0.737 | 1.667 | 3.382 | 7.185 |
| ALA/CIT | 20.930 | 4.460 | 5.779 | 6.587 | | 19.336 | 37.563 | 47.842 | 47.860 | 62.797 |
| ARG/ALA | 0.065 | 0.003 | 0.005 | 0.006 | | 0.059 | 0.136 | 0.180 | 0.199 | 0.254 |
| ARG/ORN | 0.254 | 0.009 | 0.013 | 0.017 | | 0.222 | 0.569 | 0.834 | 0.908 | 1.457 |
| ARG/PHE | 0.418 | 0.020 | 0.029 | 0.036 | | 0.365 | 0.907 | 1.235 | 1.398 | 1.991 |
| CIT/ARG | 1.396 | 0.198 | 0.281 | 0.317 | | 0.867 | 4.272 | 9.193 | 12.572 | 18.811 |
| CIT/ORN | 0.220 | 0.041 | 0.056 | 0.064 | | 0.196 | 0.427 | 0.617 | 0.706 | 0.943 |
| CIT/PHE | 0.366 | 0.090 | 0.113 | 0.126 | | 0.319 | 0.717 | 1.068 | 1.322 | 1.803 |
| CIT/TYR | 0.227 | 0.036 | 0.049 | 0.055 | | 0.195 | 0.484 | 0.722 | 0.877 | 1.117 |
| GLY/PHE | 7.704 | 1.578 | 2.321 | 2.694 | | 7.414 | 12.410 | 15.347 | 17.021 | 20.011 |
| LEU+ILE+PRO-OH/ALA | 0.445 | 0.151 | 0.190 | 0.212 | | 0.418 | 0.710 | 0.991 | 1.218 | 1.479 |
| LEU+ILE+PRO-OH/PHE | 2.752 | 0.895 | 1.173 | 1.313 | | 2.680 | 4.055 | 4.816 | 5.055 | 5.706 |
| LEU+ILE+PRO-OH/TYR | 1.641 | 0.390 | 0.536 | 0.615 | | 1.630 | 2.467 | 2.989 | 3.332 | 4.072 |
| MET/CIT | 0.977 | 0.076 | 0.116 | 0.140 | | 0.837 | 2.136 | 2.926 | 2.686 | 4.673 |
| MET/LEU+ILE+PRO-OH | 0.110 | 0.013 | 0.020 | 0.025 | | 0.103 | 0.194 | 0.260 | 0.273 | 0.491 |
| MET/PHE | 0.289 | 0.040 | 0.053 | 0.064 | | 0.278 | 0.492 | 0.612 | 0.639 | 1.091 |
| MET/TYR | 0.172 | 0.016 | 0.027 | 0.034 | | 0.162 | 0.307 | 0.401 | 0.408 | 0.753 |
| ORN/ARG | 7.638 | 0.687 | 1.015 | 1.199 | | 4.497 | 24.529 | 57.744 | 80.860 | 111.670 |
| ORN/CIT | 5.663 | 1.060 | 1.425 | 1.621 | | 5.106 | 10.788 | 15.552 | 17.746 | 24.460 |
| ORN/PHE | 1.880 | 0.304 | 0.428 | 0.500 | | 1.679 | 3.711 | 4.948 | 5.619 | 6.485 |
| PHE/TYR | 0.624 | 0.167 | 0.214 | 0.241 | | 0.602 | 0.976 | 1.255 | 1.446 | 2.231 |
| PRO/CIT | 12.219 | 1.577 | 2.270 | 2.647 | | 11.148 | 24.041 | 31.892 | 33.863 | 44.959 |
| PRO/PHE | 3.700 | 0.956 | 1.397 | 1.588 | | 3.494 | 5.960 | 7.694 | 8.435 | 10.900 |
| SA/MET | 0.068 | 0.011 | 0.016 | 0.018 | | 0.054 | 0.152 | 0.279 | 0.344 | 0.490 |
| SA/PHE | 0.016 | 0.003 | 0.005 | 0.005 | | 0.015 | 0.027 | 0.036 | 0.045 | 0.069 |
| SA/TYR | 0.010 | 0.002 | 0.003 | 0.003 | | 0.009 | 0.018 | 0.025 | 0.030 | 0.044 |
| TYR/CIT | 6.077 | 0.895 | 1.188 | 1.385 | | 5.140 | 13.101 | 18.143 | 19.049 | 27.660 |
| TYR/PHE | 1.810 | 0.449 | 0.718 | 0.797 | | 1.660 | 3.108 | 4.141 | 4.497 | 5.976 |
| VAL/PHE | 2.748 | 1.017 | 1.253 | 1.381 | | 2.703 | 3.883 | 4.609 | 5.109 | 5.747 |
| ((LEU+ILE+PRO-OH)+VAL)/(PHE+TYR) | 2.013 | 0.614 | 0.835 | 0.918 | | 2.017 | 2.802 | 3.318 | 3.771 | 4.534 |
| (C0+C2+C3+C16+C18+C18:1)/CIT | 3.317 | 0.260 | 0.439 | 0.548 | | 3.036 | 6.513 | 8.610 | 8.887 | 12.136 |
| C3/MET | 0.129 | 0.009 | 0.019 | 0.024 | | 0.110 | 0.274 | 0.430 | 0.493 | 0.782 |
