# Supplementary figures and images for: A preliminary investigation of amino acid and acylcarnitine levels in neonates from the Tibet autonomous
Source: Front Genet. 2022 Sep 26;13:941938. doi: 10.3389/fgene.2022.941938 (PMC9589887; doi:10.3389/fgene.2022.941938)

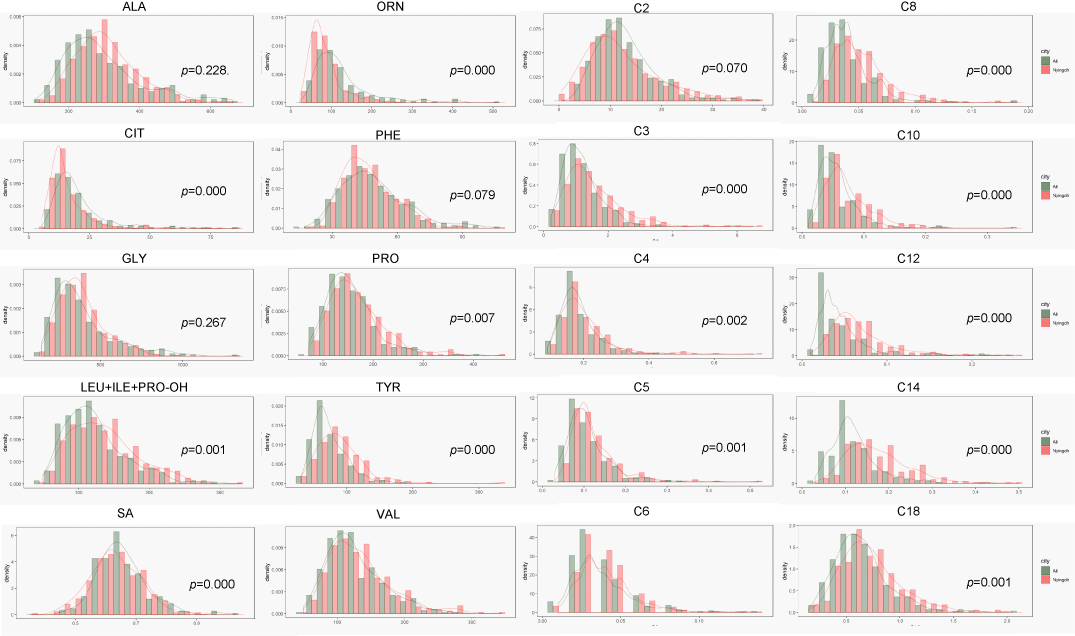

Supplement: Supplementary file 2 [file Image1.TIF]
